# Circulating selectins as potential biomarkers for sarcopenia: a case-control study

**DOI:** 10.3389/fmed.2026.1805444

**Published:** 2026-03-25

**Authors:** Hu Zhang, Huaqing Liu, Yanhui Tang, Jiatong Wu, Mingyang Du, Yanhua Song, Nannan Wang, Jianfeng Wang

**Affiliations:** 1 Department of Endoscopy, Nanyang Central Hospital, Nanyang, Henan, China; 2 Department of Gastroenterology and General Surgery, Nanyang Central Hospital, Nanyang, Henan, China

**Keywords:** biomarkers, E-selectin, L-selectin, older adults, P-selectin, sarcopenia

## Abstract

**Background:**

This study investigates the association between circulating E-selectin, L-selectin, and P-selectin levels and sarcopenia in older adults.

**Methods:**

We conducted a case-control study of 325 older adults (261 controls, 64 sarcopenia cases). Sarcopenia was diagnosed using Asian Working Group for Sarcopenia 2019. Serum selectin concentrations were measured by ELISA. Diagnostic utility was assessed via ROC curve analysis, and associations were examined using logistic regression models.

**Results:**

Serum P-selectin was significantly elevated in sarcopenic participants compared to controls (19.76 ± 10.07 vs. 13.12 ± 7.39 ng/mL, *p* < 0.001), whereas E-selectin and L-selectin levels were comparable between groups. P-selectin demonstrated discriminative capacity for sarcopenia (AUROC = 0.693) with an optimal cutoff of 17.28 ng/mL. Participants with high P-selectin (>17.28 ng/mL) exhibited increased odds of sarcopenia (multivariate OR = 4.20, 95% CI: 1.82–9.69, *p* < 0.001), low appendicular muscle mass (OR = 2.31, 95% CI: 1.44–3.73, *p* = 0.001), and low handgrip strength (OR = 1.64, 95% CI: 1.02–2.63, *p* = 0.043).

**Conclusion:**

Circulating P-selectin represents a novel, independent biomarker for sarcopenia, reflecting endothelial activation and inflammatory pathways underlying muscle wasting.

## Introduction

Sarcopenia, a progressive and multifactorial geriatric syndrome characterized by the age-related decline in skeletal muscle mass and function, represents a burgeoning public health challenge and a pivotal determinant of adverse outcomes in older adults (1, 2). Its escalating prevalence with advancing age imposes a substantial burden of frailty, falls, institutionalization, and mortality, thereby profoundly diminishing quality of life and functional independence (3). While traditional etiological factors—including nutritional deficiency, physical inactivity, and hormonal alterations—are well-recognized contributors, accumulating evidence implicates dysregulated inflammatory and proteolytic signaling as central mechanisms underpinning muscle wasting (4–7). Consequently, the identification of reliable circulating biomarkers that reflect these pathophysiological cascades has emerged as a research priority to enable early detection, risk stratification, and targeted therapeutic intervention (8).

Chronic low-grade inflammation, often termed “inflammaging,” constitutes a critical physiological driver of sarcopenia pathogenesis (9). Pro-inflammatory cytokines such as interleukin-6 (IL-6) and tumor necrosis factor-alpha (TNF-*α*) initiate a catabolic milieu that accelerates muscle protein degradation while suppressing anabolic signaling, satellite cell function, and myogenic regeneration (10, 11). While these established biomarkers primarily reflect systemic inflammatory status, they do not specifically capture vascular pathology. Concurrently, microvascular endothelial dysfunction, manifesting as impaired perfusion and altered vascular permeability, disrupts the delicate niche required for effective muscle repair and contributes to the progressive loss of muscle mass (12–15). However, circulating markers of endothelial activation remain largely unexplored in sarcopenia despite their potential to provide mechanistic insights distinct from conventional inflammatory indices. These interrelated processes underscore the necessity of investigating inflammatory and endothelial activation pathways to uncover novel biomarkers that capture the systemic nature of sarcopenia.

While several circulating biomarkers—including C-reactive protein, interleukin-6, vitamin D, and creatinine-based indices—have been associated with sarcopenia risk and severity, these primarily reflect nutritional status, renal function, or systemic inflammation rather than specific vascular pathology. Notably, despite accumulating evidence implicating microvascular endothelial dysfunction in impaired muscle perfusion and regenerative capacity, circulating markers of endothelial activation have not been systematically evaluated in sarcopenia. This represents a significant research gap, as endothelial-specific biomarkers may offer distinct mechanistic insights and complementary diagnostic utility beyond conventional inflammatory indices.

Selectins comprise a family of C-type lectin adhesion molecules—E-selectin, L-selectin, and P-selectin—that orchestrate the initial tethering and rolling of leukocytes on activated endothelium, representing a rate-limiting step in inflammatory cell recruitment (16). E-selectin is expressed *de novo* on cytokine-activated endothelial cells, P-selectin is rapidly translocated from Weibel-Palade bodies to endothelial and platelet surfaces, while L-selectin is constitutively expressed on leukocytes and is shed upon activation (17–19). The soluble forms of these molecules, measurable in peripheral blood, serve as established surrogate markers of systemic endothelial activation and leukocyte engagement (20, 21). Given that sustained leukocyte infiltration can inflict direct myofiber damage, promote interstitial fibrosis, and perturb regenerative homeostasis, dysregulated selectin-mediated pathways may plausibly contribute to the progressive muscle attrition and impaired functional capacity observed in sarcopenia (22–25).

Despite the recognized role of inflammation in sarcopenia, the specific association between circulating selectin levels and sarcopenia remains unexplored. To our knowledge, no prior study has systematically evaluated E-selectin, L-selectin, and P-selectin as integrated biomarkers of sarcopenia in older cohorts. We hypothesized that elevated concentrations of these adhesion molecules would reflect heightened inflammatory and endothelial activation, thereby correlating with key sarcopenia components and overall disease status. Therefore, this case-control study aims to investigate the relationship between serum levels of the three selectins and sarcopenia, as defined by the Asian Working Group for Sarcopenia (AWGS) criteria, in a cohort of older adults (26). Furthermore, we sought to assess their independent diagnostic utility and discriminative performance, thereby establishing a foundation for their potential integration into clinical geriatric assessment protocols.

## Methods

### Study design and participants

This case-control study was conducted at the Department of Gastroenterology, Nanyang Central Hospital, in strict accordance with the Declaration of Helsinki and was approved by the hospital’s Ethics Committee (ID: 20200105). All participants provided written informed consent, and their personal data were anonymized to ensure confidentiality. Between April 2020 and April 2025, we consecutively recruited eligible older adults aged 65 years and above who were admitted to our department and agreed to participate. Individuals with active malignancies, acute inflammatory diseases, or incomplete data on sarcopenia assessment and relevant biomarkers were excluded to preserve the integrity of the study cohort.

### Data collection

Systematic acquisition of baseline parameters was accomplished through meticulous review of institutional electronic health records. The dataset encompassed fundamental demographic descriptors (chronological age and biological sex), anthropometric indices (height and weight for BMI derivation), and lifestyle factors (smoking history and alcohol consumption). Comorbidity burden was quantified using the validated Charlson Comorbidity Index (27). Admission laboratory profiles comprised hematological indices (RBC count and hemoglobin), metabolic markers (fasting glucose), and nutritional status indicators (serum albumin). Supplemental clinical evaluations included electrocardiographic and chest radiographic assessments.

### Selectin measurement

Fasting venous blood samples were collected in the morning following overnight admission using standard aseptic technique. Serum was isolated by centrifugation at 1,500×*g* for 15 min at 4 °C and stored at −80 °C until analysis. Serum concentrations of E-selectin, L-selectin, and P-selectin were quantified using commercially available enzyme-linked immunosorbent assay (ELISA) kits specific for each human adhesion molecule, following the manufacturers’ protocols (E-selectin, #ab303747, Abcam; L-selectin, #ab314373, Abcam; P-selectin, #ab100631, Abcam). Microplates pre-coated with monoclonal capture antibodies were incubated with diluted serum samples, followed by sequential addition of biotinylated detection antibodies, horseradish peroxidase-conjugated streptavidin, and tetramethylbenzidine substrate. Absorbance was measured spectrophotometrically at 450 nm with wavelength correction at 570 nm. Concentrations were determined by interpolation from standard curves generated using serial dilutions of known standards. All samples were assayed in duplicate, with intra-assay and inter-assay coefficients of variation maintained below 5 and 10%, respectively. Laboratory personnel performing the assays were blinded to participants’ sarcopenia status.

### Sarcopenia assessment

Sarcopenia was diagnosed according to the Asian Working Group for Sarcopenia (AWGS) 2019 criteria (26), which require the presence of both low handgrip strength (HGS) and reduced appendicular skeletal muscle mass (ASM). Low HGS was defined as <28 kg for men and <18 kg for women, measured using a calibrated Jamar hydraulic dynamometer; participants performed three maximal isometric contractions with each hand, and the highest value was recorded. ASM was assessed via bioelectrical impedance analysis (BIA) using the InBody BWA2.0 device, which employs multi-frequency impedance (1 kHz to 3 MHz) across four limb segments. ASM index (ASM/height^2^) thresholds for sarcopenia were <7.0 kg/m^2^ in men and <5.7 kg/m^2^ in women. All measurements were performed by trained technicians following standardized protocols.

### Statistical analysis

Continuous variables were expressed as mean ± standard deviation. Categorical variables were presented as frequencies and percentages. Between-group comparisons were performed using independent Student’s *t*-tests or Mann–Whitney U tests for continuous data based on distribution normality assessed by the Shapiro–Wilk test, and Chi-squared or Fisher’s exact tests for categorical variables. Receiver operating characteristic (ROC) curve analysis evaluated the discriminative ability of each selectin for sarcopenia, low HGS, and low ASM, with optimal cutoff values determined by maximizing the Youden index. Participants were stratified into high and low selectin groups based on these cutoffs. Univariate and multivariate logistic regression models were constructed to assess associations between selectin levels (both continuous and dichotomized) and sarcopenia outcomes, adjusting for age, sex, BMI, CCI, and ALB as clinically relevant covariates (age and sex for multivariate model 1; age, sex, BMI, CCI, and ALB for multivariate model 2). Linear regression analyses examined relationships between selectins and continuous HGS or ASM values. Power analysis was conducted using Cohen’s method, with a medium effect size (Cohen’s d = 0.5) specified for detecting differences between sarcopenia and control groups. Two-sided *p*-values <0.05 were considered statistically significant. All statistical analyses were conducted using R software (version 4.1.2; R Foundation for Statistical Computing, Vienna, Austria).

## Results

### General information

**Figure 1:**
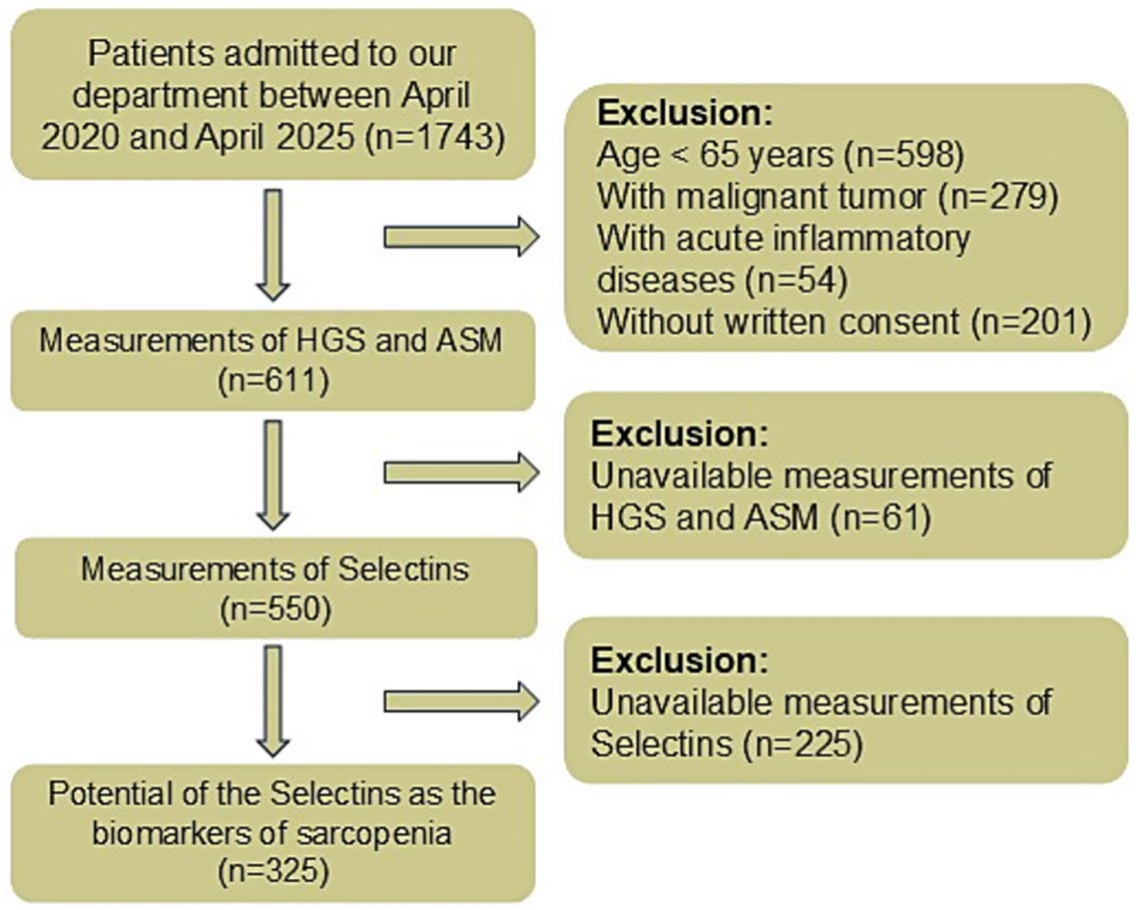
Flow chart of participant selection.

From 1,743 patients admitted to our department between April 2020 and April 2025, 611 eligible individuals aged ≥65 years remained after exclusions for malignancy, acute inflammatory conditions, and lack of consent. Following further exclusion of 61 participants with unavailable muscle measurements and 225 with insufficient serum samples for selectin analysis, 325 subjects were included in the final analytic cohort, comprising 261 non-sarcopenic controls and 64 sarcopenic cases (Figure 1).

Table 1 summarizes baseline features. The overall sample exhibited a mean age of 76.65 ± 8.49 years and BMI of 20.22 ± 6.18 kg/m^2^. No significant intergroup differences emerged for age, BMI, smoking or alcohol history, comorbidity burden (CCI > 4), cardiovascular risk factors, or laboratory parameters including hemoglobin, albumin, and glucose (all *p* > 0.05). Notably, sex distribution differed markedly (*p* < 0.001), with females comprising 61.69% of the normal group versus 28.12% of the sarcopenia group. Among the three selectins, only P-selectin demonstrated significant elevation in sarcopenic participants (19.76 ± 10.07 ng/mL) compared to controls (13.12 ± 7.39 ng/mL; *p* < 0.001), while E-selectin and L-selectin concentrations remained comparable between groups (*p* = 0.711 and *p* = 0.458, respectively).

**Table 1:** Baseline characteristics of study participants overall and by sarcopenia status.

Variables	Overall	Normal	Sarcopenia	*p* value
	(*n* = 325)	(*n* = 261)	(*n* = 64)	
Age (years)	76.65 ± 8.49	76.51 ± 8.31	77.20 ± 9.25	0.725
BMI (kg/m^2^)	20.22 ± 6.18	20.40 ± 6.28	19.52 ± 5.75	0.431
Sex (female)	179 (55.08%)	161 (61.69%)	18 (28.12%)	<0.001
Smoking history (yes)	55 (16.92%)	48 (18.39%)	7 (10.94%)	0.154
Alcoholism history (yes)	41 (12.62%)	33 (12.64%)	8 (12.50%)	0.975
CCI score (>4)	105 (32.31%)	86 (32.95%)	19 (29.69%)	0.617
Electrocardiogram (abnormal)	80 (24.62%)	61 (23.37%)	19 (29.69%)	0.293
Chest radiograph (abnormal)	58 (17.85%)	47 (18.01%)	11 (17.19%)	0.878
Hypertension (yes)	100 (30.77%)	78 (29.89%)	22 (34.38%)	0.486
RBC (10^12^/L)	4.37 ± 1.43	4.31 ± 1.41	4.61 ± 1.49	0.375
Hb (g/L)	86.43 ± 26.20	86.87 ± 26.32	84.63 ± 25.79	0.426
ALB (g/L)	35.98 ± 13.73	36.03 ± 13.73	35.78 ± 13.84	0.87
GLU (mmol/L)	5.31 ± 1.85	5.25 ± 1.82	5.53 ± 1.94	0.242
E-Selectin (pg/mL)	423.21 ± 249.72	420.92 ± 246.86	432.53 ± 262.90	0.711
L-Selectin (ng/mL)	16.71 ± 10.11	17.01 ± 10.39	15.51 ± 8.85	0.458
P-Selectin (ng/mL)	14.42 ± 8.40	13.12 ± 7.39	19.76 ± 10.07	<0.001

### Serum selectin levels and muscle parameter associations

**Figure 2:**
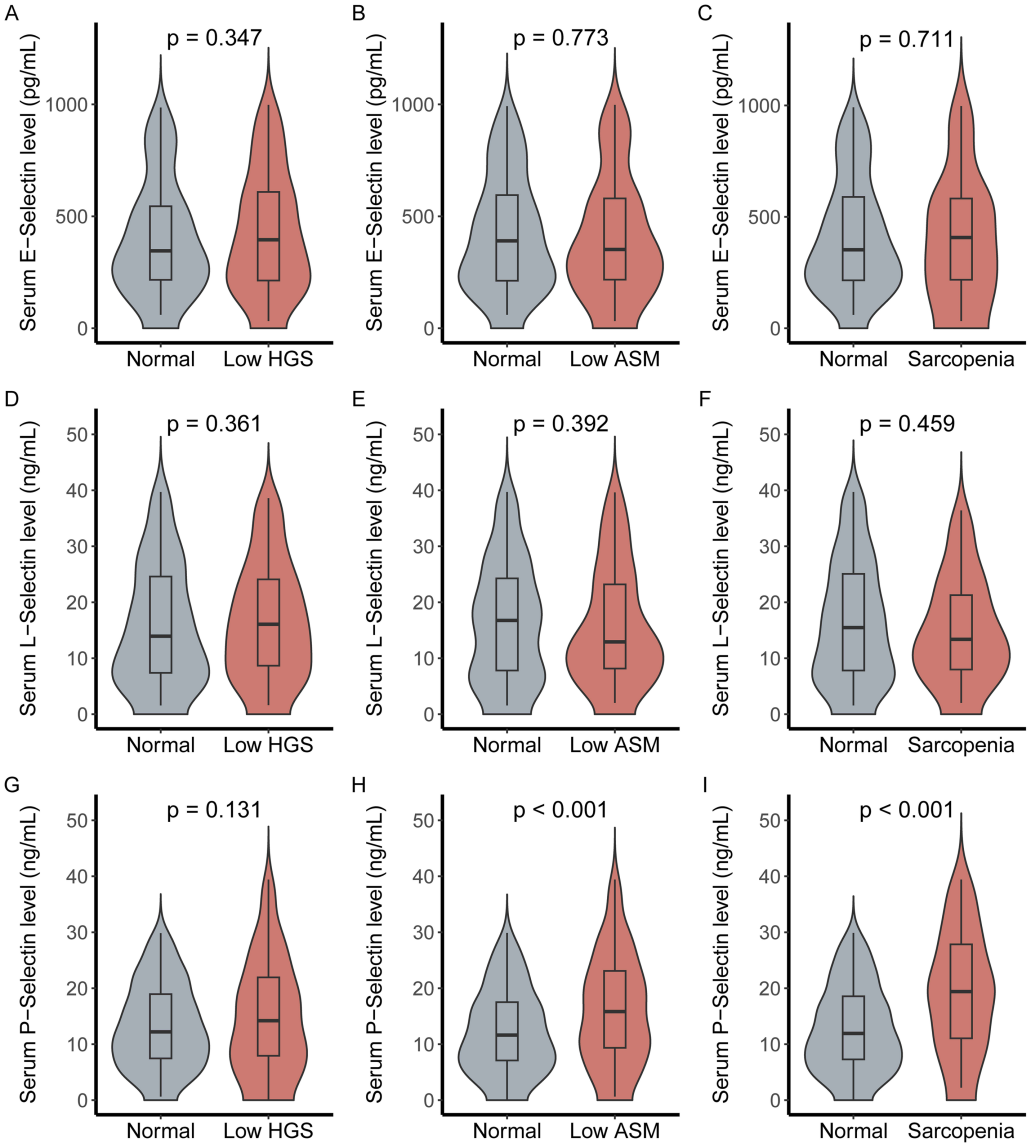
Serum selectin levels across sarcopenia trait categories. **(A–C)** E-selectin concentrations in normal participants versus those with low HGS, low ASM, and sarcopenia. **(D–F)** L-selectin concentrations across the same groups. **(G–I)** P-selectin concentrations across groups.

Following categorization by AWGS sarcopenia components, serum selectin profiles were compared across groups (Figure 2). E-selectin concentrations remained comparable between participants with normal parameters and those exhibiting low HGS (*p* = 0.711), low ASM (*p* = 0.347), or sarcopenia (*p* = 0.773). Similarly, L-selectin levels showed no significant differences across these categories (all *p* > 0.05). In contrast, P-selectin concentrations were significantly elevated in individuals with low ASM (*p* < 0.001) and sarcopenia (*p* < 0.001) relative to their respective normal counterparts, while the increase in the low HGS group did not reach statistical significance (*p* = 0.131).

Given the significant sex imbalance between groups (Table 1), we conducted sex-stratified analyses to evaluate whether the association between selectins and sarcopenia components differed by sex. As shown in Supplementary Figures 1, 2, P-selectin concentrations were significantly elevated in sarcopenic females and males compared to their respective controls, with similar patterns observed for low ASM. These findings suggest that the association between P-selectin and sarcopenia is consistent across sexes, supporting the robustness of our primary results.

Table 2 presents linear regression analyses examining relationships between selectins and continuous muscle parameters. E-selectin and L-selectin demonstrated no significant associations with HGS or ASM in either univariate or multivariate models (all *p* > 0.05). P-selectin exhibited a notable negative correlation with ASM in univariate analysis (*β* = −0.052, *p* = 0.003), persisting after adjustment for age and sex (*β* = −0.050, *p* = 0.004). However, no significant relationship emerged between P-selectin and HGS (multivariate *β* = −0.087, *p* = 0.305).

**Table 2:** Univariate and multivariate linear regression models examining associations between serum selectins and continuous muscle parameters.

Variables	HGS				ASM			
	Univariate		Multivariate		Univariate		Multivariate	
	β	*p*	*β*	*p*	*β*	*p*	*β*	*p*
E-Selectin (continuous)	−0.001	0.719	−0.001	0.66	0.001	0.902	0.001	0.825
L-Selectin (continuous)	−0.054	0.437	−0.053	0.447	0.005	0.719	0.006	0.671
P-Selectin (continuous)	−0.098	0.243	−0.087	0.305	−0.052	0.003	−0.05	0.004

### ROC curve analysis and group comparisons

**Figure 3:**
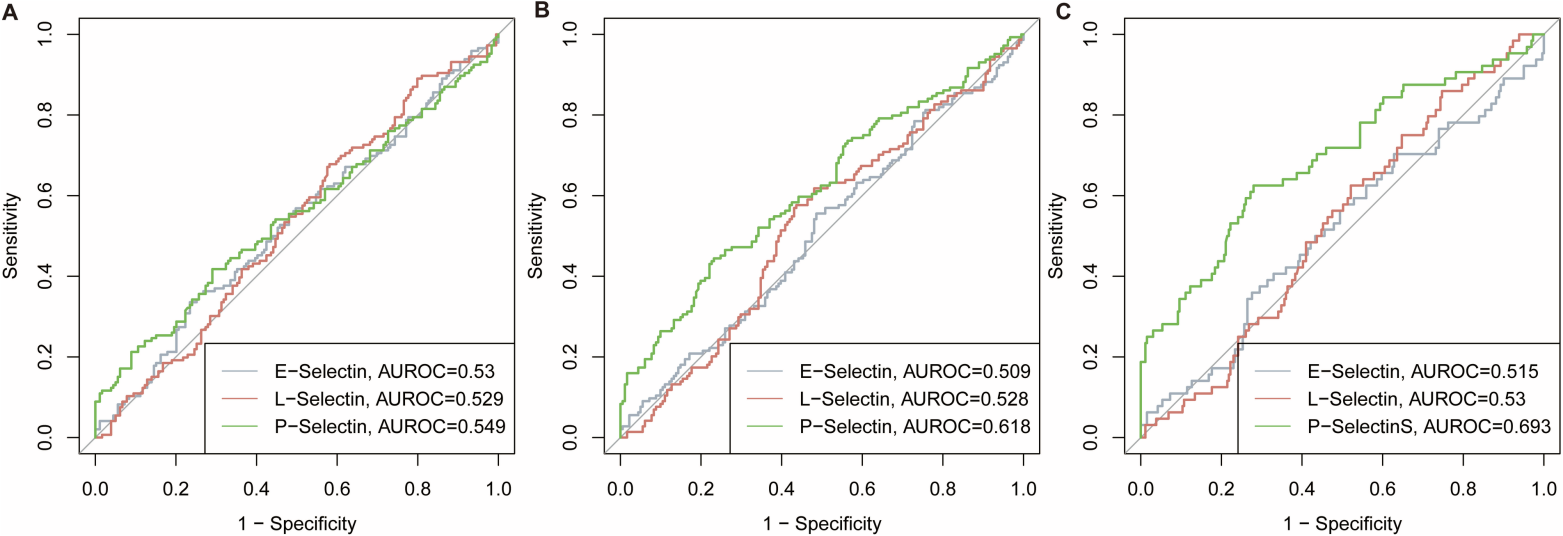
Receiver operating characteristic (ROC) curves for selectin levels predicting sarcopenia components. **(A)** ROC curves for low HGS prediction. **(B)** ROC curves for low ASM prediction. **(C)** ROC curves for sarcopenia diagnosis.

ROC curve analysis demonstrated limited discriminative performance for E-selectin and L-selectin, with AUROCs ranging from 0.509 to 0.533 across all sarcopenia components (Figure 3). However, P-selectin exhibited modestly superior discriminatory capacity, achieving AUROCs of 0.549 for low HGS, 0.618 for low ASM, and 0.693 for sarcopenia.

Based on optimal cutoffs derived from the Youden index (E-selectin: 541.48 pg./mL; L-selectin: 25.05 ng/mL; P-selectin: 17.28 ng/mL), participants were stratified into high and low selectin groups for further comparison (Table 3). E-selectin stratification revealed no significant differences in HGS, ASM, or sarcopenia prevalence between high and low groups (all *p* > 0.05). Similarly, L-selectin groups showed comparable muscle parameters, though the high L-selectin cohort demonstrated a trend toward lower sarcopenia frequency (12.00% vs. 22.00%, *p* = 0.056). In contrast, P-selectin stratification revealed significant disparities consistent with its superior ROC performance. The high P-selectin group exhibited markedly reduced ASM (6.40 ± 2.73 vs. 7.14 ± 2.57 kg/m^2^, *p* = 0.017) and elevated proportions of low HGS (53.98% vs. 40.09%, *p* = 0.023), low ASM (58.41% vs. 36.79%, *p* < 0.001), and sarcopenia (35.40% vs. 11.32%, *p* < 0.001) compared to the low P-selectin group.

**Table 3:** Sarcopenia-related outcomes stratified by high versus low selectin groups based on ROC-derived optimal cutoffs.

Outcomes	Overall	High E-selectin	Low E-selectin	*p*	High L-selectin	Low L-selectin	*p*	High P-selectin	Low P-selectin	*p*
	(*n* = 325)	(*n* = 95)	(*n* = 230)		(*n* = 75)	(*n* = 250)		(*n* = 113)	(*n* = 212)	
HGS (kg)	24.99 ± 12.73	24.39 ± 13.09	25.23 ± 12.60	0.587	25.72 ± 12.74	24.77 ± 12.75	0.571	23.71 ± 12.90	25.67 ± 12.62	0.186
ASM (kg/m^2^)	6.88 ± 2.65	6.82 ± 2.69	6.91 ± 2.64	0.785	6.99 ± 2.64	6.85 ± 2.65	0.684	6.40 ± 2.73	7.14 ± 2.57	0.017
Low HGS	146 (44.92%)	50 (52.63%)	96 (41.74%)	0.073	31 (41.33%)	115 (46.00%)	0.476	61 (53.98%)	85 (40.09%)	0.023
Low ASM	144 (44.31%)	42 (44.21%)	102 (44.35%)	0.982	30 (40.00%)	114 (45.60%)	0.392	66 (58.41%)	78 (36.79%)	<0.001
Sarcopenia	64 (19.69%)	23 (24.21%)	41 (17.83%)	0.188	9 (12.00%)	55 (22.00%)	0.056	40 (35.40%)	24 (11.32%)	<0.001

### Logistic regression analysis

Building upon these stratified findings, we conducted logistic regression analyses to quantify the independent associations between selectin levels and sarcopenia outcomes (Table 4). In univariate models, continuous P-selectin demonstrated significant positive associations with low ASM (OR = 1.059 per unit increase, 95% CI: 1.030–1.089, *p* < 0.001) and sarcopenia (OR = 1.098, 95% CI: 1.062–1.138, *p* < 0.001), while showing a marginal association with low HGS (OR = 1.029, 95% CI: 1.003–1.057, *p* = 0.032). Neither E-selectin nor L-selectin exhibited significant univariate relationships with any muscle parameter (all *p* > 0.05). The dichotomized analysis revealed that low P-selectin status (≤17.28 ng/mL) was associated with substantially reduced odds of low ASM (OR = 0.415, 95% CI: 0.259–0.659, *p* < 0.001) and sarcopenia (OR = 0.233, 95% CI: 0.130–0.410, *p* < 0.001), reinforcing the directional consistency of findings.

**Table 4:** Univariate and multivariate logistic regression analyses of selectin levels predicting sarcopenia components.

Variables	Low HGS		Low ASM		Sarcopenia	
	OR [95%CI]	*p*	OR [95%CI]	*p*	OR [95%CI]	*p*
Univariate model						
E-Selectin (continuous)	1.000 [1.000, 1.001]	0.313	1.000 [0.999, 1.001]	0.783	1.000 [0.999, 1.001]	0.739
L-Selectin (continuous)	1.008 [0.986, 1.030]	0.493	0.990 [0.969, 1.012]	0.389	0.985 [0.957, 1.012]	0.289
P-Selectin (continuous)	1.029 [1.003, 1.057]	0.032	1.059 [1.030, 1.089]	<0.001	1.098 [1.062, 1.138]	<0.001
Low E-Selectin	0.645 [0.398, 1.042]	0.073	1.006 [0.622, 1.632]	0.982	0.679 [0.383, 1.224]	0.19
Low L-Selectin	1.209 [0.719, 2.051]	0.476	1.257 [0.747, 2.140]	0.392	2.068 [1.012, 4.686]	0.06
Low P-Selectin	0.571 [0.359, 0.903]	0.017	0.415 [0.259, 0.659]	<0.001	0.233 [0.130, 0.410]	<0.001
Multivariate model 1						
E-Selectin (continuous)	1.001 [1.000, 1.002]	0.206	1.000 [0.999, 1.001]	0.922	1.000 [0.999, 1.001]	0.588
L-Selectin (continuous)	1.010 [0.988, 1.033]	0.375	0.991 [0.969, 1.013]	0.431	0.987 [0.959, 1.015]	0.372
P-Selectin (continuous)	1.024 [0.997, 1.053]	0.083	1.055 [1.027, 1.086]	<0.001	1.095 [1.058, 1.137]	<0.001
Low E-Selectin	0.634 [0.384, 1.043]	0.074	1.012 [0.620, 1.659]	0.962	0.682 [0.374, 1.260]	0.214
Low L-Selectin	1.231 [0.719, 2.126]	0.452	1.270 [0.747, 2.182]	0.38	2.178 [1.040, 5.033]	0.051
Low P-Selectin	0.611 [0.379, 0.983]	0.043	0.433 [0.268, 0.693]	0.001	0.238 [0.129, 0.429]	<0.001
Multivariate model 2						
E-Selectin (continuous)	1.001 [1.000, 1.002]	0.126	1.000 [0.999, 1.001]	0.784	1.000 [0.999, 1.001]	0.576
L-Selectin (continuous)	1.010 [0.988, 1.034]	0.366	0.991 [0.969, 1.013]	0.421	0.987 [0.958, 1.015]	0.364
P-Selectin (continuous)	1.024 [0.996, 1.053]	0.09	1.056 [1.027, 1.087]	<0.001	1.095 [1.057, 1.136]	<0.001
Low E-Selectin	0.598 [0.359, 0.991]	0.047	1.043 [0.634, 1.722]	0.869	0.672 [0.365, 1.254]	0.204
Low L-Selectin	1.228 [0.713, 2.133]	0.461	1.277 [0.750, 2.198]	0.372	2.201 [1.048, 5.098]	0.048
Low P-Selectin	0.593 [0.365, 0.959]	0.033	0.434 [0.268, 0.697]	0.001	0.232 [0.125, 0.419]	<0.001

Multivariate adjustment for age and sex (Model 1) attenuated the association between continuous P-selectin and low HGS to borderline significance (OR = 1.024, 95% CI: 0.997–1.053, *p* = 0.083). However, the relationships with low ASM (OR = 1.055, 95% CI: 1.027–1.086, *p* < 0.001) and sarcopenia (OR = 1.095, 95% CI: 1.058–1.137, *p* < 0.001) persisted robustly. The dichotomized low P-selectin variable remained significantly protective against low HGS (OR = 0.611, 95% CI: 0.379–0.983, *p* = 0.043), low ASM (OR = 0.433, 95% CI: 0.268–0.693, *p* = 0.001) and sarcopenia (OR = 0.238, 95% CI: 0.129–0.429, *p* < 0.001) after multivariate correction. E-selectin and L-selectin, in both continuous and dichotomized forms, showed no independent associations with any outcome in adjusted models (all *p* > 0.05). Collectively, these data identify P-selectin as the sole selectin family member exhibiting a consistent, dose-dependent relationship with sarcopenia and its constituent deficits.

To evaluate the robustness of our findings to additional confounder adjustment, we constructed an expanded multivariable model (Model 2) further adjusting for BMI, Charlson Comorbidity Index, and serum albumin. As presented in Table 4, the associations between P-selectin and sarcopenia outcomes remained statistically significant and largely unchanged. These results suggest that the observed relationships are independent of nutritional status, comorbidity burden, and body composition.

## Discussion

This case-control study provides the first systematic evaluation of circulating selectins as potential biomarkers for sarcopenia in older adults. Our principal finding demonstrates that P-selectin, but not E-selectin or L-selectin, exhibits a strong and independent association with sarcopenia as defined by AWGS criteria. Specifically, serum P-selectin concentrations were significantly elevated in sarcopenic individuals, demonstrated modest discriminatory capacity (AUROC = 0.693), and maintained a dose-dependent relationship with both low appendicular skeletal muscle mass and composite sarcopenia diagnosis after adjustment for confounders. These results identify P-selectin as a promising endothelial activation marker that may reflect pathophysiological processes underlying muscle wasting, while suggesting that E-selectin and L-selectin have limited utility in sarcopenia assessment.

The pathophysiology of sarcopenia involves complex interplay between inflammatory signaling, proteolytic degradation, and impaired regenerative capacity (28). P-selectin, stored in Weibel-Palade bodies of endothelial cells and *α*-granules of platelets, is rapidly mobilized to the cell surface upon inflammatory stimulation, where it mediates the initial tethering and rolling of leukocytes—a critical step in leukocyte recruitment to sites of tissue injury (29, 30). However, we acknowledge that serum P-selectin measurements cannot distinguish between endothelial and platelet-derived sources, and platelet activation may independently contribute to circulating levels. In the context of skeletal muscle, persistent low-grade inflammation characteristic of aging may trigger sustained P-selectin expression, facilitating inappropriate leukocyte infiltration that promotes myofiber damage, interstitial fibrosis, and disruption of the satellite cell niche (31–33). This proposed mechanistic link remains speculative; we cannot exclude that subclinical cardiovascular disease or microvascular dysfunction may confound the observed association, given shared pathophysiological pathways between endothelial dysfunction and muscle decline. Our observation that P-selectin correlates specifically with ASM rather than HGS may reflect its particular involvement in structural muscle remodeling and mass accretion rather than direct neuromuscular function, though this interpretation is hypothetical and requires validation through mechanistic studies incorporating muscle perfusion assessments and comprehensive cardiovascular profiling.

The lack of association between sarcopenia and E-selectin or L-selectin, despite their shared role in leukocyte adhesion, highlights important distinctions in their regulatory kinetics and pathophysiological relevance. E-selectin requires *de novo* transcription following cytokine stimulation, potentially rendering it less responsive to the subacute inflammatory milieu of sarcopenia compared to the rapidly deployable P-selectin (34, 35). L-selectin, constitutively expressed on leukocytes and proteolytically shed upon activation, may represent a more transient inflammatory signal that does not accumulate sufficiently to reflect chronic muscle catabolism (29, 36, 37). These divergent expression patterns suggest that selectin family members are not interchangeable inflammatory markers, and that P-selectin specifically captures the sustained endothelial activation most relevant to sarcopenia progression.

From a translational perspective, the diagnostic performance of P-selectin merits cautious interpretation. The AUROC of 0.693 indicates only modest discriminative capacity, and the determined optimal cutoff exhibited limited sensitivity and specificity in our cohort. While the four-fold increased odds of sarcopenia in the high P-selectin group is statistically significant, the moderate AUC value suggests that P-selectin alone is insufficient for reliable clinical diagnosis. Its potential utility, if any, may lie in serving as a complementary biomarker within multi-marker panels or as an initial risk stratification tool in resource-limited settings where bioimpedance analysis is unavailable, rather than as a standalone diagnostic test. Further validation in larger, prospective cohorts is essential before considering any clinical implementation.

Several limitations require acknowledgment. The cross-sectional design precludes establishing causality or temporal relationships between P-selectin elevation and sarcopenia development. We cannot exclude the possibility of reverse causation, wherein sarcopenia-associated pathophysiological changes, such as altered body composition, reduced physical activity, or metabolic dysregulation, may themselves contribute to elevated P-selectin levels through mechanisms including endothelial dysfunction and chronic inflammation. Longitudinal cohort studies are warranted to clarify the temporal sequence and bidirectional relationships between selectin levels and muscle decline. Our single-center cohort of hospitalized older adults may not represent community-dwelling populations, potentially limiting generalizability. Hospitalized patients frequently exhibit acute stress responses, elevated inflammatory status, and metabolic perturbations that may elevate selectins independently of chronic age-related muscle decline. Future validation in ambulatory, community-based cohorts is essential to determine whether these findings translate to non-hospitalized populations with differing inflammatory milieu. The relatively modest sample size, particularly the sarcopenia group (*n* = 64), may constrain statistical power for detecting subtle associations and subgroup analyses, though post-hoc calculations indicate adequate power (94.7%) for detecting medium effect sizes. Moreover, we used BIA to measure the muscle mass of patients. BIA, while endorsed by the Asian Working Group for Sarcopenia for clinical screening, may be less accurate than DXA in hospitalized patients with altered hydration status, potentially introducing measurement variability in our muscle mass estimates. Additionally, an important limitation is the lack of comprehensive inflammatory profiling. We did not measure established inflammaging markers such as CRP, interleukin-6 IL-6, or TNF-*α*, which are well-characterized mediators of muscle catabolism. Consequently, we cannot determine whether P-selectin reflects a distinct endothelial-specific pathway or merely correlates with systemic inflammatory burden. Future studies should integrate selectin measurements with broad inflammatory panels to disentangle their specific contributions to sarcopenia pathophysiology and evaluate their additive utility in multi-marker risk prediction models.

In conclusion, this study pioneers the investigation of selectin biomarkers in sarcopenia and establishes P-selectin as a novel circulating indicator associated with disease status. Its relationship with muscle mass, independent of confounders, suggests a potential link between endothelial activation and sarcopenia pathophysiology. However, given the moderate discriminative performance (AUROC = 0.693) and cross-sectional design, these findings should be considered preliminary. P-selectin may contribute to multi-marker risk stratification approaches pending validation in larger, longitudinal studies. These results warrant further investigation to determine whether P-selectin can serve as a component of predictive biomarker panels in the context of age-related muscle decline.

## Data Availability

The raw data supporting the conclusions of this article will be made available by the authors, without undue reservation.
