# Supplementary material for: Circulating selectins as potential biomarkers for sarcopenia: a case-control study
Source: Front Med (Lausanne). 2026 Mar 25;13:1805444. doi: 10.3389/fmed.2026.1805444 (PMC13057553; doi:10.3389/fmed.2026.1805444)
Supplement: Supplementary file 1 [file Data_Sheet_1.PDF]

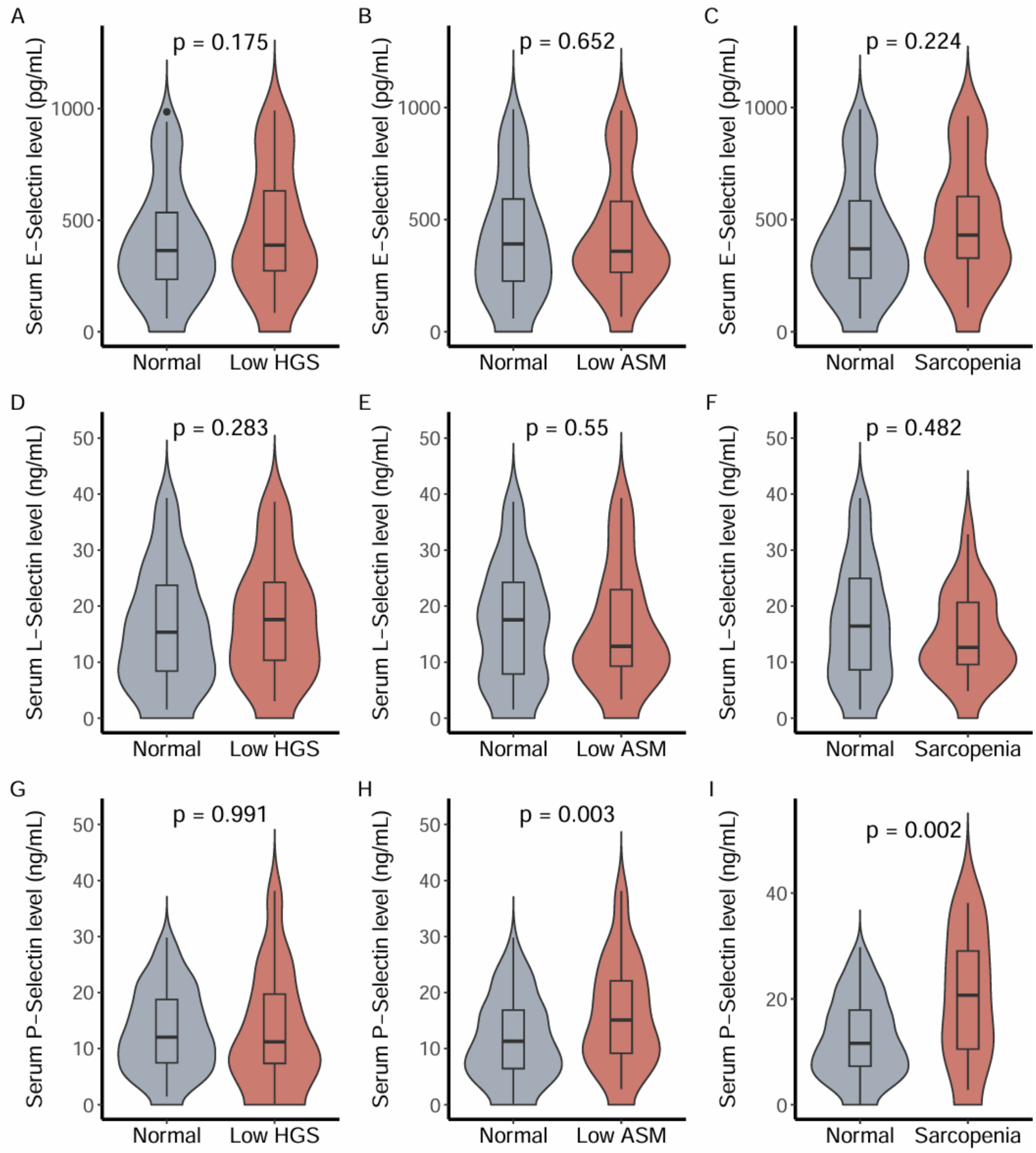


Supplementary Figure 1. Serum selectin levels across sarcopenia trait categories in females. (A-C) E-selectin concentrations in normal participants versus those with low HGS, low ASM, and sarcopenia. (D-F) L-selectin concentrations across the same groups. (G-I) P-selectin concentrations across groups.

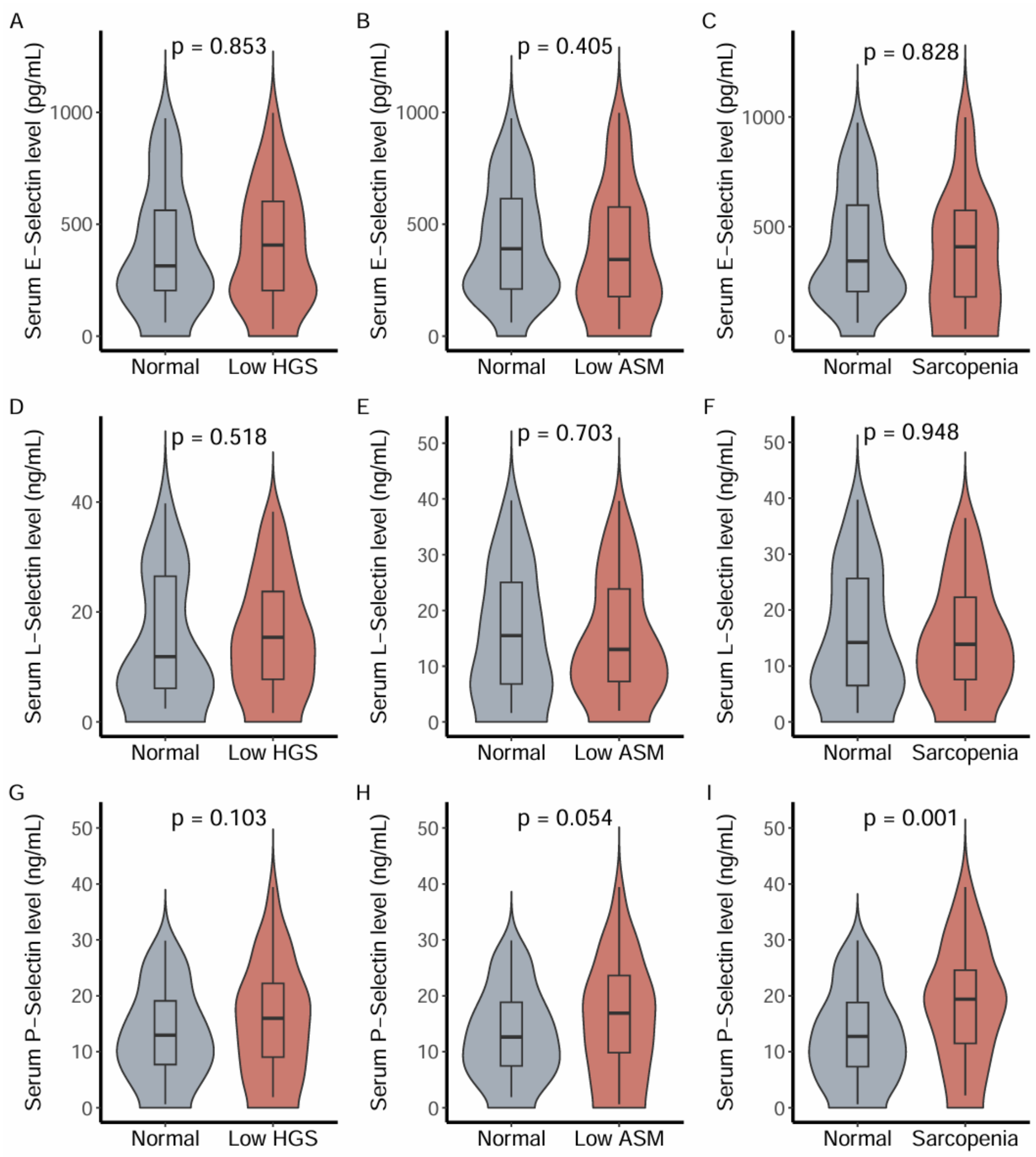


Supplementary Figure 2. Serum selectin levels across sarcopenia trait categories in males. (A-C) E-selectin concentrations in normal participants versus those with low HGS, low ASM, and sarcopenia. (D-F) L-selectin concentrations across the same groups. (G-I) P-selectin concentrations across groups.
